# Dengue virus serotype infection specifies the activation of the unfolded protein response

**DOI:** 10.1186/1743-422X-4-91

**Published:** 2007-09-24

**Authors:** Indira Umareddy, Olivier Pluquet, Qing Yin Wang, Subhash G Vasudevan, Eric Chevet, Feng Gu

**Affiliations:** 1 Novartis Institute for Tropical Diseases, 10-Biopolis Road, #05-01 Chromos, 138670, Singapore; 2 Team AVENIR, GREF INSERM U899, IFR66, Université Victor Segalen Bordeaux 2, 146 rue Léo Saignat, 33076 Bordeaux, France

## Abstract

**Background:**

Dengue and Dengue hemorrhagic fever have emerged as some of the most important mosquito-borne viral diseases in the tropics. The mechanisms of pathogenesis of Dengue remain elusive. Recently, virus-induced apoptosis mediated by the Unfolded Protein Response (UPR) has been hypothesised to represent a crucial pathogenic event in viral infection. In an attempt to evaluate the contribution of the UPR to virus replication, we have characterized each component of this signalling pathway following Dengue virus infection.

**Results:**

We find that upon Dengue virus infection, A549 cells elicit an UPR which is observed at the level of translation attenuation (as visualized by the phosphorylation of eIF2alpha) and activation of specific pathways such as nuclear translocation of ATF-6 and splicing of XBP-1. Interestingly, we find that specific serotype of virus modulate the UPR with different selectivity. In addition, we demonstrate that perturbation of the UPR by preventing the dephosphorylation of the translation initiation factor eIF2alpha using Salubrinal considerably alters virus infectivity.

**Conclusion:**

This report provides evidence that Dengue infection induces and regulates the three branches of the UPR signaling cascades. This is a basis for our understanding of the viral regulation and conditions beneficial to the viral infection. Furthermore, modulators of UPR such as Salubrinal that inhibit Dengue replication may open up an avenue toward cell-protective agents that target the endoplasmic reticulum for anti-viral therapy.

## Background

**Figure 1:**
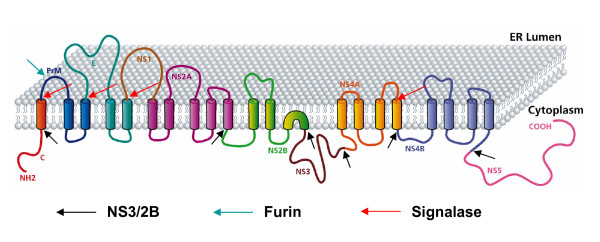
**Dengue viral polyprotein and its predicted membrane topology. **Schematic representation of the membrane topology of the proteins and their cleavage by host (red and blue arrows) or viral (black arrows) proteases. The 11 kb genome of Dengue is translated into a single polypeptide and this polyprotein traverses the ER membrane at several positions. prM, E, NS1 and a part of NS4A and NS4B are thought to localise to the ER lumen via hydrophobic signal sequences whereas the remaining proteins are thought to be localized on the cytoplasmic side of the ER membrane.

Dengue virus (DENV) is a member of the *Flaviviridae *family, which include West Nile virus (WNV), yellow fever virus, Japanese encephalitis virus (JEV), and tick-borne encephalitis virus (TBEV), among others [1]. Dengue is caused by four antigenically distinct viruses designated as Dengue virus type 1–4 (DENV 1–4) and is transmitted between vertebrate hosts by insect vectors. The most serious manifestations of the infection are Dengue hemorrhagic fever (DHF) and Dengue shock syndrome (DSS). No effective vaccine or antiviral drug therapy is currently available against Dengue viruses. The genome of Dengue virus consists of a single stranded, non segmented, positive sense ribonucleic acid (RNA) of about 11 kb in length [1]. The genome is translated into a single polypeptide which is co- and post-translationally processed by host signalases as well as the virus encoded serine protease into the three structural and seven non structural proteins (NS) in the order C-prM-E-NS1-NS2A-NS2B-NS3-NS4A-NS4B-NS5 that traverse the Endoplasmic Reticulum (ER) membrane (Fig. 1). Dengue and other flaviviruses are thought to replicate in the cytoplasm, mature on intracellular membranes and egress by exocytosis and in some cases by budding at the plasma membrane [2]. The host ER is the primary site of envelope glycoprotein biogenesis, genomic replication, and particle assembly of flaviviruses. In the course of productive infection, flaviviruses induce proliferation and hypertrophy of the ER membranes [3-5]. Moreover, a large amount of flaviviral proteins are synthesized in infected cells, thus overwhelming the ER folding capacity. As a natural consequence, we hypothesize that these events will lead to the activation of the ER stress response which in turn will modulate various signaling pathways resulting in cell survival or death decisions.

In mammalian cells, the ER stress response, also called the Unfolded Protein Response (UPR) is mediated by three transmembrane proteins that act as sensors: i) the protein kinase-like ER resident kinase (PERK), ii) the activating transcription factor 6 (ATF6) and iii) the inositol-requiring enzyme 1 (IRE1) [6]. The activation of PERK and IRE1 is driven by their oligomerization followed by their trans-auto phosphorylation. Activated PERK phosphorylates the eukaryotic initiation factor eIF2α thus resulting in translation attenuation. This is also accompanied by the activation of negative feed-back transcriptional loops. This includes the up-regulation of the pro-apoptotic mRNA CHOP and that encoding GADD34 whose association with the phosphatase PP1 leads to the dephosphorylation of eIF2α [6]. Activated IRE-1 cleaves an unconventional 26-nucleotides intron from X-Box binding Protein-1 (XBP-1) mRNA which leads to a change in the open reading frame and leads to the translation of an active transcription factor [7]. The spliced form encoded XBP-1 protein (sXBP-1) is involved in the transcriptional activation of a number of genes including the ER mannosidase-like protein EDEM which is involved in protein degradation. In parallel, upon accumulation of mis-folded proteins in the ER, ATF-6 exits this compartment to migrate to the Golgi apparatus where it is cleaved by S1P and S2P proteases [8]. ATF-6 cytosolic fragment is an active transcription factor responsible for transcriptional induction of XBP-1 as well as many ER chaperone encoding genes (reviewed in [6,9]).

Several studies have shown that in some cases virus infections activate the three branches of the UPR. For instance the UPR master regulator – BiP is induced in cells infected with Respiratory syncytial virus [10], hanta viruses [11], hepatitis C viruses [12] as well as flaviviruses such as cytopathic strains of BVDV [13] and JEV [14]. Activation of PERK has also been reported in infection with herpes simplex virus [15,16], cytomegalovirus [17] and BVDV [13]. The IRE1-XBP1 axis has been recently shown to be activated in cells infected with JEV and Dengue [18] whereas the ATF-6 pathway has been reported to be activated upon HCV infection [19]. It is also becoming increasingly evident that many viruses have evolved mechanisms to cope with UPR response or to utilize it to their benefit. Indeed, the herpes simplex virus genome encodes a GADD34 homolog – γ_1_34.5 protein which leads to the dephospholyation of eIF2α and overcomes the PERK response [15,20]. The African swine fever virus overcomes the transcriptional activation of CHOP induced by Thapsigargin [21] and cytomegalovirus overcomes translation inhibition despite activation of eIF2α phosphorylation [22].

In recent years it has become clear that the ability of viruses to regulate cellular responses to infection is a key determinant for the physiological consequences of infection. Since the activation of the UPR is on the one hand essential for cell survival during viral infection and on the other hand detrimental to viral replication, it is therefore thought that a balance between the two would determine the outcome of the infection in host cells. Consequently, it may be advantageous for viruses to modulate the UPR to its advantage. For instance, replication of hepatitis C virus has been shown to stimulate the ATF6 pathway [19], but attenuate the IRE1-XBP1 pathway [19]. Since UPR induction upon Dengue infection is currently under-investigated, the aim of this study was to determine the UPR characteristics under those circumstances. The understanding of such response would also yield critical information to control Dengue infection.

## Results

### Dengue infection induces phosphorylation of eIF2α

**Figure 2:**
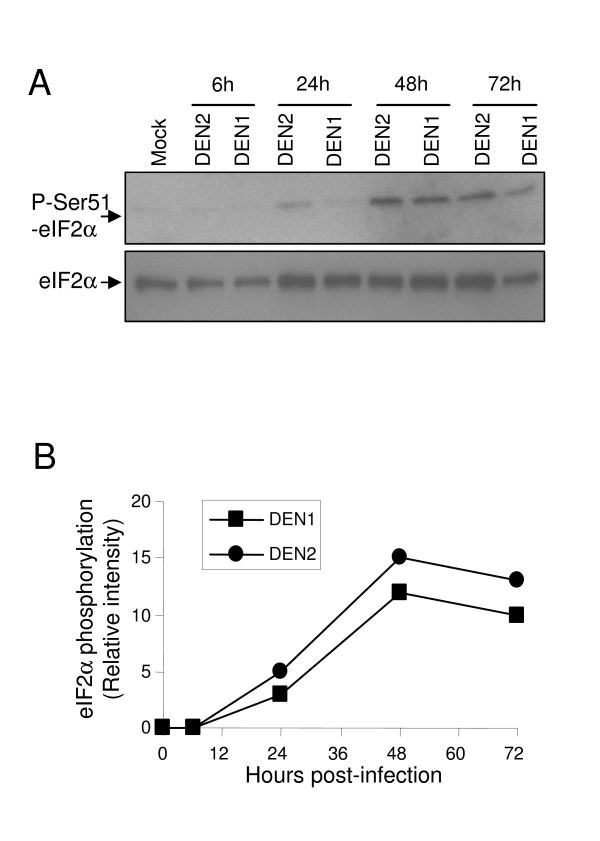
**Dengue infection induces phosphorylation of eIF2α**. **(A) **A549 cells were infected with DENV1 or DENV2 viruses at 10 MOI and lysed at indicated time points in lysis buffer. Protein extracts were subjected to immunoblot analysis with anti-phosphoserine51-eIF2α (top panel), and anti-eIF2α (bottom panel) antibodies. **(B) **Phosphoserine51-eIF2α levels from (A) were quantified and normalized to eIF2α levels and plotted.

Upon virus infection, eukaryotic cells respond in part by shutting-down translation. This process is mediated by the phosphorylation of the translation initiation factor eIF2α [23]. To determine whether eIF2α is phosphorylated upon Dengue infection, A549 cells were infected for 6 to 72 hours with DENV2 (TSV01) or DENV1 (MY 10245) viruses and harvested at indicated time points post-infection (Fig. 2). Cell lysates were first analyzed by immunoblot using an antibody against phospho-eIF2α. In both cases of Dengue infection, phosphorylated forms of eIF2α were detected at 24 h post-infection, and accumulated until 72 h (Fig. 2A). This indicates that eIF2α kinases such as PERK or PKR are activated upon infection of A549 cells by Dengue virus. Interestingly, by using an antibody against total eIF2α we showed that eIF2α protein expression levels increased at 24 h post-infection and remained elevated up to 72 h compared to mock-infected cells (Fig. 2A), while thapsigargin (TG, our ER stress positive control) treatment alone did not modify total eIF2α protein levels (data not shown). This result suggests that Dengue virus might be able to overcome or compensate the UPR response by inducing more elF2α protein for translation. This is further supported by a recent study which showed that translation is not attenuated by Dengue infection [24] although eIF2α is phosphorylated. The overall ratio of phospho elF2α and elF2α is quantified for both DENV 1 and DENV 2 infection (Fig. 2B). The two serotypes of Dengue showed similar pattern of peak phosphorylation at 48 hours, with DENV2 infection slightly stronger than DENV1.

### Dengue infection promotes GADD34 expression

**Figure 3:**
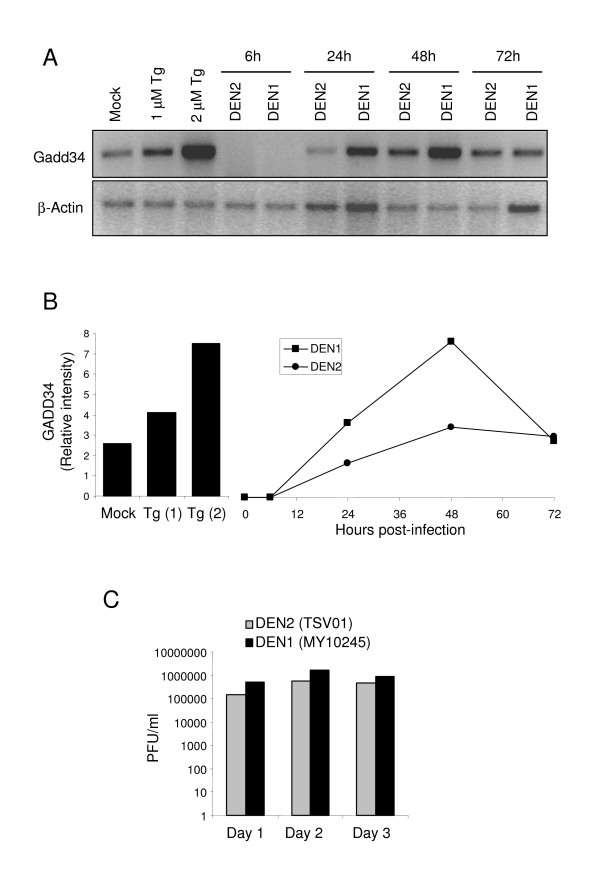
**Dengue infection induces the upregulation of GADD34 mRNA**. **(A) **A549 cells were either treated with increasing concentration of thapsigargin (TG) for 1 hour or infected with DENV2 or DENV1 viruses (10 MOI) at indicated time points. GADD34 mRNA (top panel) and β-actin mRNA (bottom panel) levels were determined by semi-quantitative RT-PCR with specific primers (see Materials and Methods). **(B) **Densitometric quantification of GADD34 mRNA levels from (A) were normalized to β-actin mRNA levels and plotted in histograms (TG) or graphs (DEN1 and 2). The ratio of the GADD34 to β-actin of the uninfected (Mock) sample was considered as basal level (0 hour) and negative value was also represented as basel level. **(C) **A549 cells were infected with either 1 moi of DENV1 (MY10245) or DENV2 (TSV01). Virus production after Day 1, Day 2 and Day 3 of infection were quantified by plaque assay and expressed by PFU/ml.

As a recent study showed that translation is not attenuated by Dengue infection [24] although eIF2α is phosphorylated, we consequently asked whether the regulatory subunit of protein phosphatase (GADD34) that dephospholyates eIF2α was specifically induced upon Dengue infection. To this end, A549 cells were infected as before and RNA extracted for RT PCR analysis to measure GADD34 mRNA expression. TG, a well-recognized inducer of ER stress, served as a positive control in these assays (Fig. 3A). GADD34 mRNA expression levels were quantified and normalized to actin mRNA levels (Fig. 3B). When compared to mock-infected cells, Dengue infection induced the expression of GADD34 at 24 hours post-infection (Fig. 3A and 3B) most likely to compensate for the induction of eIF2α phosphorylation. Interestingly, infection by DENV1 was a more potent inducer of GADD34 expression than DENV2 (Fig. 3A and 3B). In order to test whether this was caused by different replication of the two viruses, we compare the growth of the two viruses by plaque assay. Figure 3C shows that the DENV2 (MY10245) strain indeed produced slightly more virus at each time point than the DENV1 (TSV01) strain, but the two viruses grew at similar rate in A549 cells. Furthermore, it was noted that at 6 h post-infection, GADD34 mRNA expression level was lower than uninfected. This result may be explained by a virus-induced consumption of GADD34 mRNA through a translation-dependent process. These data suggested that Dengue infection led to increased elF2α phosphorylation, as well as the activation of downstream feedback loop controlling its dephosphorylation, in a strain dependent manner.

### Dengue infection activates the ATF6 pathway

**Figure 4:**
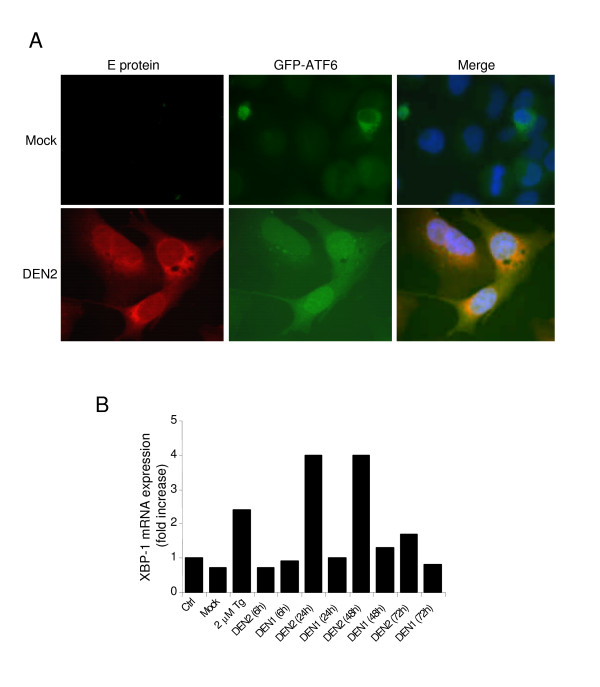
**Dengue infection activates the ATF6 pathway**. **(A) **A549 cells were transiently transfected with GFP-ATF6 plasmid. After 24 h, the cells were left untreated or infected with DENV2 virus at 10 MOI. Twenty four hours post-infection, immunocytochemistry analysis was performed to detect GFP-ATF6 (green), the viral E protein (red), and cell nuclei were detected by DAPI staining (blue). **(B) **A549 cells were either treated with thapsigargin (TG) or infected with DENV1 and DENV2 and total mRNA was extracted and analyzed for XBP1 expression by RT-PCR as in Figure 5. The XBP1 mRNA level was quantified by densitometry as the total of all spliced forms of XBP1 and expressed as fold increase compared to untreated cells (Ctrl).

We next examined whether the ATF6 pathway was activated upon Dengue infection. In response to ER stress, ATF6 exits the ER to traffic to the Golgi apparatus, where it is processed to its active form which then translocates to the nucleus. We assessed the presence of this nuclear form of ATF6 as a marker of its activation during infection by Dengue virus. To this end, GFP tagged ATF6 was transfected into A549 cells which then were infected with 10 moi of DENV2 (NGC) 24 hours after transfection. The cells were fixed in cold methanol and immuno-fluorescence was performed using anti-E protein antibodies. ATF6 localized to cytoplasmic ER-like structures in mock infected cells whereas an intense signal was observed in the nucleus in DENV2 infected cells. These results suggest that Dengue virus activates the ATF6 pathway of the UPR. We next confirmed ATF6 activation by assessing ATF6-dependent transcriptional activation of XBP1 gene [7,25]. We measured total XBP1 mRNA in Dengue-infected cells and TG-treated cells by RT-PCR (Fig. 4B). The level of XBP1 mRNA significantly increased in Dengue-infected cells at 24 h post-infection. We noticed that DENV2 infection showed a higher effect on XPB1 mRNA than DENV1. As expected, TG treatment led to a 3 fold increase in the amount of XBP1 mRNA compared to control, thus confirming ATF6 activation upon Dengue infection. In addition we detected the XBP-1 hybrid form as previously reported [26,27].

### Dengue infection activates the XBP1 pathway

**Figure 5:**
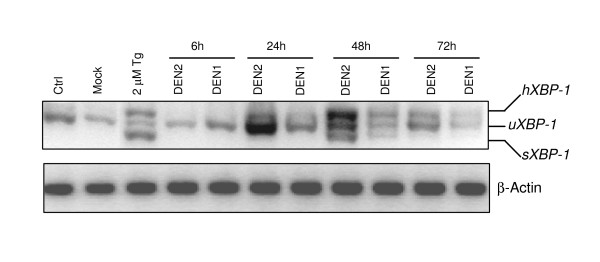
**XBP1 is spliced in Dengue infected A549 cells**. A549 cells were either treated with thapsigargin (TG) or infected with DENV2 and DENV1 (10 MOI) and harvested at indicated time points. Total mRNA was extracted and analyzed with XBP1 primers (top panel) or β-actin primers (bottom panel) by semi-quantitative RT PCR. The PCR products were run on a 3% agarose gel and the spliced (sXBP1), unspliced (uXBP1) and the hybrid (hXBP1) forms are shown. Thapsigargin was used as a positive control for induction of XBP1 splicing (sXBP1) and β-actin mRNA levels as loading control.

Upon ER stress, IRE1 processes XBP1 mRNA to result in an unconventional splicing of a 26-nucleotides intron and a translational frame shift. The spliced form of XBP1 is translated into a transcription factor. To determine whether the IRE1 pathway is activated in DENV2 and DENV1 infected A549 cells, we analyzed the splicing of XBP1 mRNA by RT-PCR using specific primers (Fig. 5). In mock-infected cells, only the unspliced form of XBP1 mRNA (uXBP1) was detected. In Dengue infected cells, the spliced form of XBP1 mRNA (sXBP1) was detected 48 h post-infection as well as in our positive control with TG. We noticed a hybrid form of XBP1 mRNA (hXBP1) upon treatment with 2 μM TG and upon Dengue infection (24 h post-infection and thereafter) [26,27]. We also noticed that cells infected with DENV2 seemed to express higher levels of XBP1 than mock-infected cells or DENV1 infected cells. These results clearly showed that XBP1 splicing is induced by Dengue replication and that Dengue virus activates the IRE-1/XBP-1 pathway of the UPR.

### Attenuation of eIF2α dephosphorylation modulates dengue replication

**Figure 6:**
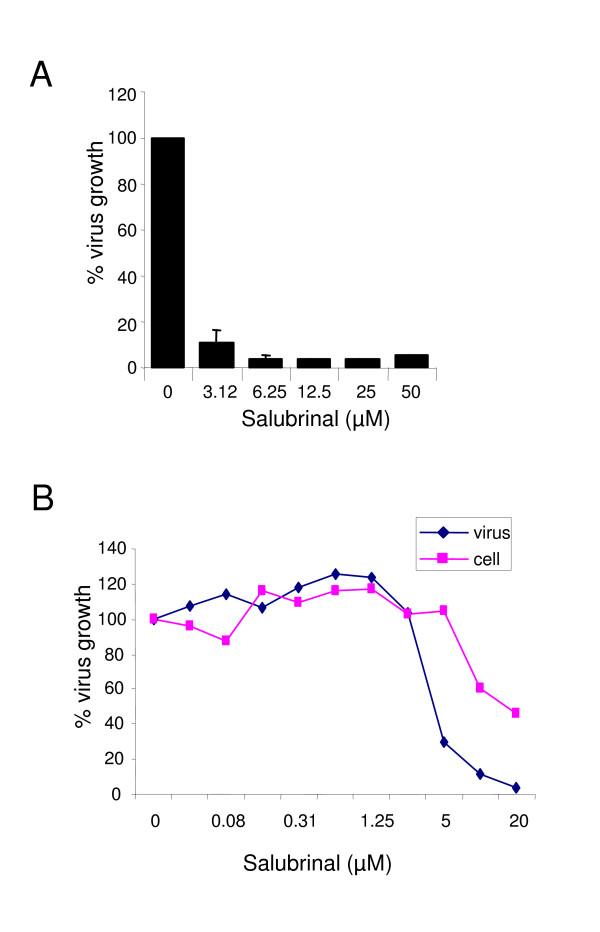
**Treatment with Salubrinal modulates Dengue viral replication**. **(A) **A549 cells were pre-treated for one hour with indicated concentrations of Salubrinal and then infected with DENV2 virus at 10 m.o.i for 48 hours. Salubrinal remained for the rest of the infection. Supernatants were collected for plaque assays. Inhibition of virus growth in the presence of salubrinal is expressed as a percentage of that in cells without salubrinal. The values represent means +/- SD from three independent experiments. **(B) **A549 cells were infected with DENV2 at 10 m.o.i and Salubrinal was added with the indicated concentrations at the time of infection for 48 hours. Viral replication (virus) was scored by immunolabeling using an anti-E antibody. Cell number (cell) was measured by propidium iodide after fixation. Percentage of viral replication and cell numbers were calculated using no salubrinal as 100%.

We have demonstrated that upon Dengue infection, all three branches of the UPR are activated as a host response. We next determined whether the modulation of the UPR could have any impact on virus growth. Salubrinal, a selective inhibitor of the protein complex (containing the protein phosphatase 1 and its cofactor GADD34) that dephosphorylates eIF2α was tested in our Dengue virus infection assay. Salubrinal has previously been reported to inhibit the replication of HSV [28]. We first tested the impact of Salubrinal on cellular toxicity. In our assay, Salubrinal was non-toxic at doses < 5 μM, CC50 (50% of cell cytotoxicity) was determined to reach approximately 10 μM in A549 cells (Fig. 6B). A549 cells were then treated with Salubrinal for one hour prior infection with DENV2 virus. Forty-eight hours later, supernatants of infection were collected and virus production was quantified by plaque assay. Addition of Salubrinal one hour prior to infection showed an eighty percent reduction of the virus at 3.12 μM (Fig 6A). Similar results were found when Salubrinal was added one hour post infection. (See Additional file 1). We then used a second method of viral growth measurement which consists of staining virus infected cells using antibodies against the Envelop protein of dengue virus (see Material and Methods, Immunolabeling Assay). Using this assay, we obtained seventy percent inhibition of the virus at 5 μM, when Salubrinal was added at the same time as infection (Fig 6B). Together, these data showed that Salubrinal reduced Dengue virus growth at micromolar concentrations when added 1 hour before, at the same time or 1 hour post-infection (salubrinal remained for the whole course of infection), thus indicating that modulation of the UPR, in this case, increase of the elF2α phosphorylation significantly reduced Dengue virus infection.

## Discussion

Many positive-strand RNA viruses need to modify intracellular membranes of their host cells in order to create a compartment suitable for virus replication [29,30]. Although this phenomenon has been well documented, little is known about the mechanisms triggered by viruses to induce intracellular membrane proliferation. An increasing amount of literature supports the hypothesis that viruses like other ER stress signals may induce membrane proliferation through the activation of specific components of the Unfolded Protein Response [31,32]. These observations are also supported by the occurrence of replication and maturation of flaviviruses in close association with the host ER and the membrane rearrangements observed in the course of flavivirus-productive infection [2]. Moreover, it has been shown that JEV [14], BVDV [13] and HCV infections [33] induce the Unfolded Protein Response. Consequently we initiated a study to characterize the UPR response to Dengue infection.

The phosphorylation of PERK has been used as an early marker for ER stress [34]. Although we attempted to determine the phosphorylation status of PERK in Dengue-infected A549 cells, we failed to see any PERK signals even for positive control using the ER stress inducers TG and DTT. Thus, the potential role of PERK activation in the Dengue induced UPR is unclear from our present study. However, microarray analyses described earlier showed that different strains of DENV2 induced the expression of PERK and PKR to a different extent (unpublished information). Moreover, we could detect phospholyation of PKR by DENV2 (data not shown) and Dengue virus induced the phosphorylation of eIF2α in A549 cells. It is therefore possible that both PKR and PERK kinases might separately phosphorylate eIF2α in response to Dengue infection.

Despite this phosphorylation event, translation is not attenuated in Dengue virus infected cells [24]. We consequently suspected that Dengue virus might activate a compensatory pathway to prevent UPR-mediated translation attenuation. Because of the deleterious effects of the host's protein synthesis inhibition, many viruses have evolved distinct mechanisms to counteract eIF2α phosphorylation as a means to avoid, at least in part, the antiviral action of interferons [35]. For instance, the γ_1_34.5 protein of herpes simplex virus is highly homologous to GADD34 and has been shown to alleviate translational arrest in cells treated with TG and DTT [15]. In our study, RT PCR analyses of GADD34 mRNA expression levels showed that Dengue induces the expression of GADD34 at approximately the same time as eIF2α phosphorylation is triggered. This led us to postulate that Dengue virus may compensate the eIF2α phosphorylation event by enhancing the expression of GADD34 which leads to enhanced dephosphorylation of eIF2α and consequently overcomes the block in translation. To corroborate our hypothesis, we analyzed the antiviral effect of Salubrinal on Dengue. Salubrinal was recently discovered as a small molecule inhibitor of the protein complex (containing the protein phosphatase 1 and its cofactor GADD34) that dephosphorylates eIF2α [28] and it has been shown to inhibit the replication of HSV [28]. Using two methods to score viral growth, we showed that Salubrinal dramatically reduced Dengue infection. We therefore conclude that enhancing eIF2α phosphorylation using Salubrinal helped the host cells to increase the translational inhibition consequently leading to reduced Dengue virus production.

The transcriptional activation of ATF6 is critical to the UPR, since ATF6 induces gene expression products necessary for protein refolding. Our data show that the ATF6 pathway is also activated by Dengue virus infection (Fig. 4A). Moreover, ATF6 has been shown to up regulate the level of XBP1 mRNA [7], which, once spliced by IRE1 plays a role in transcriptional activation. In our studies XBP1 mRNA was increased upon Dengue infection (more elevated in DENV2 infection as opposed to that of DENV1) (Fig. 4B). This suggested first that Dengue serotypes may selectively modulate ATF6 activation to either inhibit aspects that could be deleterious to the progress of the viral infection or enhance host's ability to favor it. It would therefore be exciting to study the mechanism of activation of ATF6 especially in terms of viral serotypes and strains and its effect on virus infectivity. XBP1 also plays a critical role in the UPR as it activates proteins of the degradation pathway (EDEM) that target mis-folded or unfolded proteins whereas the unspliced form results in an inactive protein. Our data show that splicing of XBP1 mRNA is triggered upon Dengue infection, consistent with the activation of the IRE1 pathway (Fig. 5). Similar results were reported earlier with JEV and DENV2 [18]. Furthermore, it was also reported that the XBP1 downstream genes such as EDEM1 and p58 (IPK) were induced in Dengue infected cells [18]. Interestingly, transactivation of XBP1 target genes are suppressed in HCV [19]. The discrepancy of XBP1 induction between these viruses might reflect the differences in infection patterns of these viruses; while HCV usually causes chronic infection, JEV and Dengue cause acute infection. It is noteworthy that knock down of XBP1 had no effect on viral production suggesting that XBP1splicing is beneficial but not essential for virus production. Nevertheless, increased cytopathic effects were noticed in XBP1 knock out cells in response to Dengue infection indicating that XBP1 alleviates ER stress induced by Dengue infection [18].

Our study is one of the first to report activation of a global UPR activation upon Dengue virus infection and has mostly focused on understanding the initial events in this process. However, the molecular mechanisms by which Dengue infection activates ER stress remain to be elucidated. In Dengue infected cells, three viral proteins are glycosylated and accumulated in the ER lumen, namely, the precursor of membrane protein (prM), the envelope protein (E), and the non-structural protein NS1 and accumulation of these in the ER may contribute to UPR induction. These and several non-structural proteins of Dengue (NS2A, NS2B, 2K-NS4B and NS2B-NS3) have been shown to induce XBP-1 splicing but none of them to the extent that whole virus is capable of [18]. Some of the flaviviral non structural proteins are hypothesized to be viroporins [36] and may cause homeostasis imbalance of calcium and other ions in the ER, thereby triggering a more extensive activation of the UPR. Moreover, during virus maturation, virions budding out from the ER appear to consume the constituents of phospholipid and sterol of the ER membrane, which may not only activate the UPR but also induce ER proliferation [14].

Initiation of the UPR is critical for cell survival and consequently for viral replication. However, prolonged/excessive UPR can lead to cell death. Therefore differential regulation of ER stress by viruses would dictate the balance between viral pathogenesis and replication. Although the pathogenesis of Dengue related disease remains poorly understood, virus-induced cell death by apoptosis may be a crucial pathogenic event [37]. It has been suggested that apoptosis is an innate defence mechanism, which allows the organism to control virus infection by elimination of infected cells through phagocytosis [38]. However, several viruses have been shown to induce apoptosis, which can be detrimental to the host [39-42]. Apoptotic cell death has been implicated as a cytopathological mechanism in response to Dengue infection both *in vitro *and *in vivo *[38,42-44]. These observations suggest that virus-induced apoptosis may contribute to the pathogenesis of Dengue. While the molecular pathways by which viruses induce apoptosis are not well understood, it is thought that apoptosis may be initiated in response to viral proteins or cellular signals and regulated by cellular proteins such as bcl-2, p53, myc, and c-fos. Several viruses also induce apoptosis mediated by ER stress. Infection of JEV exhibits severe cytopathic effects caused by CHOP and P38^MAPK ^mediated apoptosis. Tula virus infection activates the JNK pathway while BVDV activates caspase-12 to initiate apoptosis [11,13,14]. It is therefore conceivable that ER stress response to Dengue infection might play an important role in Dengue pathogenesis. Further patient-based studies with various strains of Dengue would be needed to confirm the role of virus mediated UPR in Dengue pathogenesis.

## Conclusion

This report provides evidence that Dengue infection induces and regulates the three branches of the UPR signaling cascades. This is a basis for our understanding of the viral regulation and conditions beneficial to the viral infection. Furthermore, modulators of UPR such as Salubrinal that inhibit Dengue replication may open up an avenue toward cell-protective agents that target the endoplasmic reticulum for anti-viral therapy.

## Methods

### Viruses, cell lines and constructs

DENV2 (TSV01, NGC) and DENV1 (MY 10245) were used in this study. TSV01 was used in most of the DENV2 study except when NGC was indicated. The propagation of virus was carried out in C6/36 cells utilizing RPMI-1640 medium containing 10% fetal bovine serum (FBS) (Gibco). Virus titers (plaque forming unit per ml, PFU/ml) were determined by a plaque-forming assay on BHK-21 cells as previously described. Viral infections for ER stress experiments were done on the A549 cell line propagated in F12 medium (Gibco). ATF6-GFP was constructed by PCR amplification of the full-length human ATF6α cDNA followed by Gateway^® ^(Invitrogen) cloning into pDONR201 to generate an Entry clone which was then recombined into peGFP to generate a N-terminal GFP fusion protein.

### ER stress treatment, preparation of cell lysates, and immunoblot

Cells were grown to 80% confluence. Thapsigargin (1–2 μM) was added for one hour or cells infected with Dengue virus for the indicated period of time. Cells were then washed once in phosphate-buffered saline and lysed on ice in 150 mM NaCl, 50 mM Tris-HCl, 1% Nonidet P-40, 0.25% Na deoxycholate, 1 mM Na_3_VO_4_, 50 mM NaF, and Complete protease inhibitors (Roche). Protein concentration was measured using the Bradford reagent and normalized. Equal amounts of proteins were loaded on SDS-PAGE and analyzed by immunoblot with specific antibodies.

### RNA extraction and RT PCR analysis

Total RNA was isolated using Qiashedder/Rneasy RNA purification columns (Qiagen). Reverse transcription was performed using oligodT primer (1^st ^Base, Singapore) and PCR was carried out using the primers indicated below. Commercially available β-actin primers were ordered form 1^st ^Base, Singapore. PCR products were separated by electrophoresis on a 3% agarose gel and visualized by ethidium bromide staining as previously described [26,45]. The following primers were used XBP1_F AAA CAG AGT AGC AGC TCA GAC TGC; XBP1_R TCC TTC TGG GTA GAC CTC TGG GAG, GADD34_F GTG GAA GCA GTA AAA GGA GCA G, GADD34_R CAG CAA CTC CCT CTT CCT CG.

### Reagents

Salubrinal and Thapsigargin were from Calbiochem. Anti-phospho eIF2α and eIF2α antibodies were from Cell signaling. Anti-E monoclonal (4G2) antibody was generated in-house, secondary antibody for ELISA (anti-mouse HRP) was purchased from Santacruz and secondary antibody for immunoflorescence (anti-mouse texas red) was purchased from Jackson immunoresearch.

### Immunolabeling assay

Cells were seeded on the day before infection to reach approximately 80% confluence. They were then infected with Dengue virus for 48 hours, washed in PBS, and fixed for 4 minutes in cold methanol. The fixed cells were incubated with anti-E-antibody 4G2 (hybridoma supernatant,1:20) for 1 hour and anti-mouse-HRP antibody (1:2000, Sigma) for another one hour. After washes, Tetra methyl benzidine substrate (Sigma) was added and absorbance readings at 450 nm were used to measure virus infection. Cells were then washed 3 times with PBS and Propidium iodide (125 ug/ml) was added and measured at corresponding fluorescent wave length for cell number.

### Plaque assay

BHK-21 cells were cultured in 24 well plates and incubated with virus in a serial diluted manner (10-fold) for 1 hr before media was aspirated and replaced with 0.5 ml of 0.8% methyl-cellulose medium (with 2% FBS). Plates were then incubated for 5 days before the media was removed and cells fixed in 4% formaldehyde for 20 minutes then rinsed in water and stained with crystal violet for 20 min and rinsed again. Plaques were counted manually and concentrations of plaque forming units per ml (pfu/ml) of the sample cell culture supernatant calculated.

## Competing interests

The author(s) declare that they have no competing interests.

## Authors' contributions

IU was involved in the conception and conducted the experiments described in this study as well as drafting the manuscript. QYW did some of the experiments described in Figure 3 and 6. OP actively participated to the writing. EC was responsible for initiation and conceptualization of the project and was involved with writing the manuscript. SV provided supervision. FG was responsible for the project and was involved in experiments, analysis and writing. All authors have read and approved the final manuscript.

## Supplementary Material

Additional file 1 **One hour post-treatment of Salubrinal in infection by plaque assay**. A549 cells were infected with DENV2 at 10 m.o.i for 2 days and treated with Salubrinal one hour after infection with indicated concentrations for 2 days. Supernatants were collected for plaque assays and expressed by PFU/ml. The values represent means +/- SD from three independent experiments. Click here for file
