# Supplementary figures and images for: Dengue virus serotype infection specifies the activation of the unfolded protein response
Source: Virol J. 2007 Sep 24;4:91. doi: 10.1186/1743-422X-4-91 (PMC2045667; doi:10.1186/1743-422X-4-91)

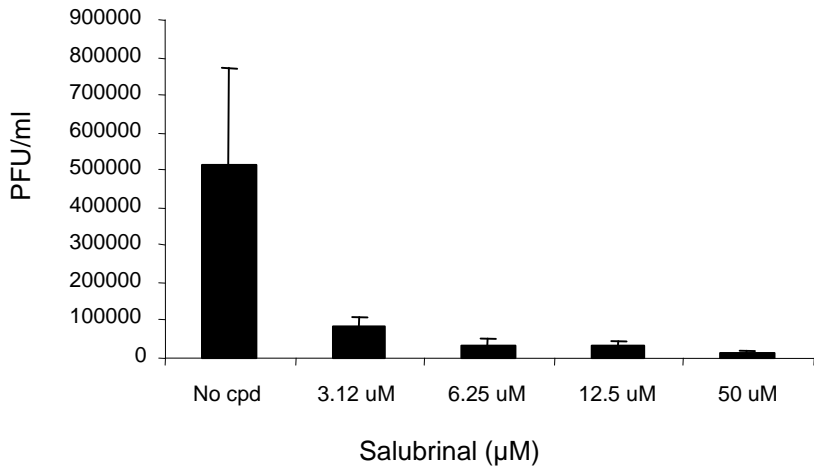

Supplement: Additional file 1 — One hour post-treatment of Salubrinal in infection by plaque assay. A549 cells were infected with DENV2 at 10 m.o.i for 2 days and treated with Salubrinal one hour after infection with indicated concentrations for 2 days. Supernatants were collected for plaque assays and expressed by PFU/ml. The values represent means +/- SD from three independent experiments. [file 1743-422X-4-91-S1.pdf]
